# North Carolina State Dental Association

**Published:** 1877-10

**Authors:** 


### ARTICLE III.

### *North Carolina State Dental Association.*

The annual meeting of the North Carolina Dental Association, commenced at Raleigh, Tuesday, September 4th 1877.

After a harmonious and interesting meeting of three days, adjourned to meet at Charlotte, N. C., 1st Tuesday in September, 1878.

The election of officers resulted as follows:

*President*—Dr. J. W. Hunter, Salem.

1*st Vice-President*—Dr. E. L. Hunter, Enfield.

2*nd Vice-President*—Dr. D. A. Robertson, Greensboro.

*Secretary*—Dr. W. H. Hoffman, Charlotte.

*Treasurer*—Dr. J. H. Crawford, Raleigh.

---

